# Urine microscopy score combined with albumin creatinine ratio score improves prediction of future acute kidney injury (AKI) and worsening AKI

**DOI:** 10.1186/cc13572

**Published:** 2014-03-17

**Authors:** JJ Dixon, K Lane, W Fleming-Nouri, H Cheema, P Walker, I MacPhee, B Philips

**Affiliations:** 1 St George's Hospital and University of London, UK; 2 St George's, University of London, UK; 3 St George's Hospital, London, UK

## Introduction

Patients with AKI have a high morbidity and mortality. Diagnosis of AKI may be improved by examination of the urinary sediment with microscopy and by measurement of urine albumin. A urine microscopy score (UMS; 0 to 4 points) of renal tubular epithelial cells and granular casts has previously been developed to aid diagnosis. We have devised a urine albumin:creatinine ratio score (ACRS; 0 to 4 points) and have combined this with the UMS with the aim of stratifying the risk of developing future AKI or worsening AKI (UMS- ACRS; 0 to 8 points). The aims were to compare UMS-ACRS in critically ill patients with and without AKI at ICU admission, and to determine whether a high UMS-ACRS can predict if critically ill patients develop AKI or worsening AKI.

## Methods

Investigators were blinded to diagnosis prior to urine collection. Microscopy was performed on centrifuged urine obtained from 227 consecutive critically ill patients in a general ICU on day 1 of admission. Five photographs were taken and the mean UMS was calculated. An independent reviewer scored the photographs. The urine albumin:creatinine ratio was calculated and the ACRS determined. The UMS was then combined with the ACRS.

## Results

Mean UMS-ACRS ± SD was higher (2.66 ± 1.57) in patients with AKI on ICU admission (*n *= 106) compared with those without AKI (mean UMS-ACRS = 2.40 ± 1.03; *n *= 120), unpaired *t *test *P *= 0.14. Patients who developed AKI or worsening AKI *(n *= 58) had a mean score of 2.79 ± 1.21 versus 2.30 ± 1.14 in those who never developed AKI or improved (*n *= 150), *P *= 0.006. UMS-ACRS score >2 on admission had a sensitivity of 0.89 for identifying progressive AKI. UMS-ACRS score of 5 to 8 on admission had a positive predictive value for worsening AKI of 60%, a negative predictive value of 69% and a likelihood ratio 3.1 for developing AKI or worsening AKI.

## Conclusion

The mean UMS-ACRS is higher in patients with AKI. Combining UMS with ACRS improves prediction and stratification of which patients will develop AKI, or progressive AKI, after ICU admission. Clinical implications are that urine microscopy and ACR calculation are safe, inexpensive, non-invasive and may improve prediction of AKI in critically ill patients, potentially leading to earlier intervention and improved outcomes.

